# Evaluation of Issues Affecting Time Between Study Completion, Manuscript Submission, Acceptance, and Publication in Medical Journals

**DOI:** 10.7759/cureus.23184

**Published:** 2022-03-15

**Authors:** Paresh G Koli, Ankita Kulkarni, Yashashri C Shetty

**Affiliations:** 1 Pharmacology and Therapeutics, Seth Gordhandas Sunderdas Medical College (GSMC) and the King Edward Memorial (KEM) Hospital, Mumbai, IND

**Keywords:** research and publication, published in pubmed journal, journal impact factor, journal citation report, journal rank

## Abstract

Introduction: Difficulty in finding the appropriate journal, adherence to the formatting differences between various journals, publication fees, delay in acceptance/rejection, etc., are a few reasons due to which much research is not published or when published the data in the research may become outdated. There are no studies to find out the issues which affect the time delay between study completion, submission to the journal, acceptance by the journal, and publication. With this background, we conducted this study.

Methods: This study was exempted by the Ethics committee as it was based on online data. Journal Citation Reports (JCR) 2020 (Clarivate analytics), CiteScore, and Google Scholar were used to sort the high-, moderate-, and low-impact factor journals. Forty-five journals each from high-, medium- and low-impact factors (h-index median, Google Scholar Metrics h5-index) were selected. Similarly, 15 predatory scientific journals were chosen. Journals with medical science backgrounds were chosen by randomization. Only original research articles were included. From each journal, five articles were chosen randomly from the latest issue pre-pandemic. The search was performed from April 2021 to June 2021. Variables analyzed were indexing of the journal, publication fees, level of impact factor, specialty domain, number of editors, frequency per year, date of study completion, date of submission, date of acceptance, date of publication, and h-index median. Data were compiled in Microsoft Excel Workbook (Microsoft, Redmond, WA, USA) and analyzed using IBM Corp. Released 2019. IBM SPSS Statistics for Windows, Version 26.0. Armonk, NY: IBM Corp. Variables of time were represented as median and interquartile range, and the number of journals and processing fees for publication were descriptively analyzed.

Results: Out of 60 journals selected, 300 original articles were analyzed. There were 26 specialty-wise journals; the commonest was multispecialty journals. The fastest time from study completion to submission, submission to acceptance, submission to publication, and acceptance to publication was 15.5, 30, 61, and 0 days, respectively, and the slowest duration was 1636, 452, 615, and 456 days, respectively. PubMed indexed journals had a higher number of editors, h5-index, and h5 median, and slower time for acceptance and publication compared to non-PubMed indexed journals (p<0.05). Predatory journals had a lower h5-index and h5 median along with faster time to acceptance and publication compared to high and moderate impact factor journals (p<0.05). Journal with faster acceptance had faster publication as well (r=0.85), but no impact of the number of editors, number of issues per year (frequency), and publication fees with time to acceptance and publication.

Conclusion: Though PubMed indexed journals with a greater number of editors and high fees are slower to publish articles but they are a safe option for researchers. The impact factor does not effect the speed of publication for non-predatory journals. Paying high fees and choosing a journal with more issues per year does not ensure quick publication to the researchers.

## Introduction

Worldwide, more than a million research articles were published in various journals in the year 2020. USA and China are the top countries from where the researchers publish their work [1]. Commonly in investigator-initiated studies, the researchers write the manuscript and send it for publication. But in sponsored studies, protocols and articles are published by the writing teams. To get the study published by a researcher, finding an appropriate journal for the study takes time, but in sponsored studies, the studies are published faster because of dedicated teams. Many papers submitted for peer review are poorly written and full of errors concerning style, grammar, punctuation, and formatting. Cramping the entire research work into meaningful 3000 words is not always possible [2]. Delay in publication can make data outdated, and the relevance of the data is lost [3].

A 2013 study by Beller et al. on systematic reviews found that the median time to acceptance of study was about five months and to publication was about eight months [4]. For clinical trials, a study showed that the median time from completion to the first submission of the main results was 10 months, and the time to publication was 23 months [5]. An analysis of 100 systematic reviews suggested that because of new research published between the conduct and publication, 7% of the reviews were out of date on the day of publication [6]. A study in 2008, before the release of the PRISMA Statement, found that the median time from the systematic review search process to publication was 61 weeks [7]. Overall, the peer review time and publication time of biomedical Indian journals included in that study seem to be long (taking about a year) [8]. There is another study regarding publication time between three Indian versus three international dermatology journals, and it found that the publication duration of Indian journals was 6-12 months [9].

Ineffective writing skills, delay in manuscript writing, difficulty in finding the appropriate journal, adherence to the formatting differences between various journals, publication fees, delay in rejection/acceptance, etc., are a few reasons much research are not published, or when published the data in the research may become outdated.

There are no studies done to assess the journal-related issues which affect the time delay between study conduct, submission to the journal, acceptance by the journal, and publication in medical science journals. With this background, we planned to do this study.

## Materials and methods

Institutional Ethics Committee exempted the study [IEC 409/2021] because the data under evaluation was freely available in the public domain. Journal Citation Reports (JCR) 2020 (Clarivate analytics), CiteScore metrics 2020, and Google Scholar were used to sort the journals in descending order of citation metrics (Eigenfactor, Impact factor, CiteScore, h5-index, etc.). A total of 50 journals, each from medical science backgrounds from high, medium, and low citation metrics were randomly chosen (top 50, middle 50, and last 50 journals were high, moderate, and low citation metrics journals). Journal not having h-index median, and Google Scholar Metrics h5-index were excluded as these are the only citation metrics indexes available for most journals. Journals not publishing original articles were excluded. After exclusions, a final list of 15 journals from each citation metric was included in the study. Fifteen predatory scientific journals were chosen by randomization from an online predatory journals list [10]. For predatory journals without the Google Scholar h5-index and h5 median scores, zero scores were given. The final list of 60 journals (15 each from high-, moderate-, low-citation metrics, and predatory) was compiled by use of randomization codes. From each journal, five articles were chosen randomly from the latest issue pre-pandemic (the year 2019). The search was performed from April 2021 to June 2021.

Variables in the studies/journals recorded were Indexing of the journal, publication fees, level of citation metrics, specialty-wise journal, number of editors working for the journal, frequency of publication per year (number of issues per year), date of study completion, date of submission, date of acceptance, date of publication, Google Scholar h-index, and h5-index median.

Data were compiled in Microsoft Excel (Microsoft, Redmond, WA, USA). Data were analyzed using IBM Corp. Released 2019. IBM SPSS Statistics for Windows, Version 26.0. Armonk, NY: IBM Corp. Variables of time were represented as median and interquartile ranges. Comparison between two groups was done by Mann-Whitney U test, and more than two groups were done by Kruskal-Wallis test with Bonferroni correction. Spearman’s correlation was applied for comparison between continuous variables. P<0.05 was considered statistically significant.

## Results

A total of 60 journals and 300 original articles were scrutinized. Out of 60 selected journals, 39 were PubMed indexed. None of the predatory journals were PubMed indexed, five low-citation metrics, and one moderate citation metrics journal was also not PubMed indexed. Median publication fees for PubMed indexed journals were $1800 (IQR 0-3440) and $63.53 (IQR 13.5-100) for non-PubMed indexed journals. Among the predatory journals, only three had Google Scholar h5-index and h5 median scores. Table 1 shows the comparison of PubMed indexed and non-PubMed indexed journals.

**Table 1:** Comparison of PubMed indexed journal with non-PubMed journals * - p< 0.05 significant by Mann-Whitney U test. Median (Interquartile range)

	PubMed Indexed	non-PubMed Indexed	p-value
No of editors	63 (50 to 95)	41 (18.75 to 64.25)	0.002*
Frequency per year	8 (6 to 12)	11 (5.5 to 12)	0.172
Publication Fees ($)	1800 (0 to 3440)	63.53 (13.5 to 100)	0.057
Google Scholar Metrics h5-index	38 (24 to 75)	0 (0 to 7)	0.000*
Google Scholar Metrics h5 median	53 (35 to 141)	0 (0 to 9)	0.000*
Study Completion to Submission (days)	511 (291 to 830)	468.5 (195.75 to 689.38)	0.419
Submission to Acceptance (days)	131 (94 to 186.5)	47 (30.5 to 81.75)	0.004*
Submission to Publication (days)	189 (139 to 277)	90.5 (52 to 168.75)	0.023*
Acceptance to Publication (days)	32 (9.5 to 73)	28.5 (17.5 to 87.25)	0.739

There were 26 specialty-wise journals found in the analysis, of which most common were multispecialty, followed by pharmacology and oncology. The median number of editors in PubMed indexed journals was higher at 61 (IQR 45.5-92) than 42 (IQR 18.5-55) for non-PubMed indexed journals (p=0.002). The median frequency of publication was 12 (IQR 6-12) for PubMed and six (IQR 5-12) for non-PubMed indexed journals (p=0.172).

The fastest time from study completion to submission, submission to acceptance, submission to publication, and acceptance to publication was 15.5, 30, 61, and 0 days, respectively, and the slowest duration was 1636, 452, 615, and 456 days, respectively. Predatory journals had a lower time to acceptance and publication compared to high and medium citation metrics journals. Table 2 shows the comparison of citation metrics.

**Table 2:** Comparison of Levels of citation metrics * - p<0.05 significant by Kruskal-Wallis test with Bonferroni correction Median (Interquartile range)

	Level of citation metrics				
	High	Medium	Low	Predatory	p-value
No of editors	70 (54 to 122)	69.5 (45.25 to 102.5)	55.5 (34 to 72.75)	43.5 (13.75 to 64.25)	0.063
Frequency per year	12 (10 to 36)	6.5 (6 to 12)	6 (4 to 12)	11 (4 to 12)	0.002* (Low vs High)
Publication Fees	3250 (2400 to 3830)	872.13 (0 to 3585)	30 (0 to 1400)	68.42 (26.69 to 500)	0.081
Google Scholar Metrics h5-index	125 (52 to 176)	34 (31 to 39)	16 (8 to 20)	0 (0 to 0)	0.000* (Predatory vs. High & Medium, Low vs. High)
Google Scholar Metrics h5 median	179 (85 to 284)	48 (45 to 59)	21(10 to 27)	0 (0 to 0)	0.000* (Predatory vs High & Medium, Low vs. High)
Study Completion to Submission (days)	802 (249 to 997)	488.25 (333.5 to 604.25)	426.25 (263 to 643.38)	405 (164.5 to 712.25)	0.468
Submission to Acceptance (days)	121 (85 to 182)	146.5 (120.25 to 174.5)	159 (77.25 to 199)	38.5 (27 to 54)	0.01* (Predatory vs. Medium)
Submission to Publication (days)	189 (170 to 208.5)	217 (129 to 462)	207.5 (126.25 to 410)	75.5 (38.5 to 96)	0.005* (Predatory vs. Medium), 0.036* (Predatory vs. Low)
Acceptance to Publication (days)	29 (11.5 to 59)	55 (9.25 to 272.25)	35.5 (11.25 to 89)	25.5 (8.75 to 44.25)	0.84

There was a positive correlation between the date of submission to publication and submission to acceptance with (r= 0.85). Other variables like publication fees, citation metrics, days to submission, acceptance, and final publication when correlated with each other were found to have moderate and weak correlations. Table 3 shows the comparison between journal factors and durations.

**Table 3:** Correlation between journal factors with durations Spearman’s rho value

	No of editors	Frequency per year	Publication Fees	Google Scholar h5-index	h-index median	Completion to submission	Submission to acceptance	Submission to publication
Frequency per year	0.07							
Publication Fees	0.05	0.36						
Google Scholar Metrics h5-index	0.33	0.42	0.33					
h-index median	0.32	0.43	0.31	1.00				
Completion to submission	-0.06	0.10	0.32	0.20	0.19			
Submission to acceptance	0.23	0.03	0.11	0.35	0.35	0.01		
Submission to publication	0.26	-0.08	-0.17	0.37	0.39	0.03	0.85	
Acceptance to Publication	0.15	-0.29	-0.38	0.02	0.05	-0.14	0.10	0.50

## Discussion

In our study, we found predatory journals had a lower duration from submission to acceptance and publication compared to medium citation metrics journals. Also, there was no association of the number of editors and frequency per year between the journals with various citation metrics.

This study was an attempt to find the reasons for the time between study completion, manuscript submission to acceptance, and publication in medical science journals. Publication plays a key role in the career of a medical science graduate. It also matters to the institution and countries to lead in publications because it brings the institution and the country into the ranking list and limelight. These institutes are preferred for research grants and admission by students. Usually, it is presumed that developed countries are well ahead in research because of the training, conducive environment for research, and promote publications. As a result, major publishers belong to these countries. China is leading, followed by the USA in publications because of the favorable research environment and funding [1].

The important objective of any published study is to bring science or evidence to fellow researchers. The publication is also meant for creating evidence, which can be used by the medical fraternity for the betterment of society. The publication is also required for getting a consultant job in a corporate hospital or getting promotions/ appraisals in the academic career. In the Indian medical context, guidelines for publication by faculty in Indian Medical schools made by the Medical Council of India (MCI)/National Medical Commission (NMC) keeps changing every few years regarding author sequence, article type, and journal indexing. If publications do not happen on time, other researchers might publish earlier in a journal with faster acceptance. Many times manuscripts are timely written, but delays might be because of the complicated submission process of journals. Delay in rejection by journals postpone the publication, which might be more relevant for the present time (e.g., COVID-19 pandemic). Many of the researchers are not good at language, editing, writing skills and don’t have time and money to hunt appropriate journals [3].

Many citation metrics like the Altmetrics, Eigenfactor, Source normalized impact per paper (SNIP), G-index, H-index, Journal-level CiteScore, Impact factor, SJR (SCImago Journal Rank), etc., are available, but not all are used by most journals. Google Scholar Metrics were chosen as it was available for most journals, and not all journals are included in a single metric. As citation metric is one of the main factors by which journals are chosen by the researchers, it indicates the quality of the research it was used to sort the journals.

Authors usually prefer publishing their articles in PubMed indexed journals for better visibility and impact [11]. Among the 45 journals selected, all had Google scholar's h5 index and h5 median index type of citation metrics, but it has its limitations [12]. PubMed indexing is one of the highest acclaimed and recognized systems in the publication area, and publishing articles in PubMed indexed journals help researchers increase outreach and visibility. Most of the journals with citation metrics available were PubMed indexed, and all predatory articles were not PubMed indexed. Predatory journals publish the articles extremely fast, with no peer review, and are based on profit. So, the data many times is not reliable or authentic [12]. Data on science should not be based on a profit model as it passes through low-quality research, which might negatively affect the lives of patients.

It was found that, on average, publication fees were lower at $68.42 (IQR 26.69-500) for predatory journals compared to high $3250 (IQR 2400-3830) and low $872.13 (0-3585) citation metrics journals. The journal charges the authors at the time of manuscript submission to fund editorial and peer review administration. If the researcher doesn’t have a sponsored project, he may choose a journal without publication charges because he cannot pay the processing fees to the journal. Many journals without publication fees have articles behind a paywall which makes their reach limited. But there was no statistically significant difference found across the journals regarding publication fees. Multispecialty was the commonest journal found in the analysis, so all the specialties articles are included in the analysis. The next specialty found in common was pharmacology since pharmacology touches all the branches and oncology. After all, recent research and publication are more in oncology because of the unmet need in this area.

In our study, PubMed indexed journals were found to have a higher no. of editors, h-5 index, h5 median, days to acceptance, and publication compared to non-PubMed indexed journals. We presumed that if there were more members on the editorial board and review board, such journal may fast-track publication, but we did not have such findings in our study. The frequency of issues per year was lower for low citation metrics journals six (IQR 4 to 12) compared to high citation metrics journals 12 (IQR 10 to 36). Our assumption that the higher the frequency of issues, the faster is the processing time was proven to be not significant in this study.

A study by Malke Asaad et al., who have analyzed the articles from Time from Submission to Publication in Plastic Surgery Journals, found the median time from submission to in print publication (TT) was 10.3 months (IQR 8 - 12.6), with a median time from submission to acceptance of 4.6 months (IQR 3 - 6.8), and from acceptance to the publication of 5.4 months (IQR 4.2 - 6.3). The finding of Median duration from submission to acceptance in the present study was less than compared to the prior study. Again, the median duration from Study Acceptance to Publication was 31 days, which was less than compared to the prior study [13]. 

According to the consensus definition, “Predatory journals and publishers are entities that prioritize self-interest at the expense of scholarship and are characterized by misleading information, deviation from best editorial and publication practices, a lack of transparency, and/or the use of aggressive and indiscriminate solicitation practices.” Predatory journals had a lower time for acceptance and publication in our study. This may be because of their model or not sending articles for peer review and accepting articles faster based on the fees paid. Also, their articles are posted online rather than printed. Online publication has decreased the time between acceptance and in-print publication, as seen in the study by Shah [8]. This might be one reason predatory journals are quicker in publication.

Low-citation metrics and predatory journals had lower h5 index and h5 median compared to high and medium citation metrics journals in our study. There was a low-to-moderate level of correlation between the h5 index and h5 median compared to the number of editors, frequency per year, and publication fees in our study. There was no statistically significant difference in the citation metrics of journals based on their reporting of submission, revision, or acceptance times of the manuscripts, as seen in a study by Chen H [14].

H-index articles give the research output based on the total number of publications and the total number of citations to those contributions of the author's works. Kaltun and Hafner mention that there are alternatives such as fractional allocation measures such as h-frac are better, and correlation of the h-index with awards that indicate recognition by the scientific community has substantially declined [15]. But as these metrics were not available for all journals, they could not be analyzed.

Overall, there were differences in journals in many of the variables analyzed but going for PubMed indexed journal is a safer option for the researchers to get the research published in good journals. Faster acceptance and publication (within a few weeks) with lower publication fees and no Google h5-index and h5 median are a deterrent to the researcher and could mean that the journal is probably a predatory journal which will bring down the impact of a good research study.

Limitations of our study were that as a single citation metric is not used by all journals, journals could not be compared based on those metrics.

## Conclusions

Though PubMed indexed journals with a greater number of editors and high fees are slower to publish articles, they are a safe option for researchers. The impact factor does not affect the speed of publication for non-predatory journals. Paying high fees and choosing a journal with more issues per year does not ensure quick publication to the researchers.
